# Links between the amount of antipsychotic medication prescribed per population at general practice level, local demographic factors and medication selection

**DOI:** 10.1186/s12888-020-02915-3

**Published:** 2020-11-07

**Authors:** A. H. Heald, M. Stedman, S. Farman, C. Khine, M. Davies, M. De Hert, D. Taylor

**Affiliations:** 1 Manchester University, The School of Medicine and Manchester Academic Health Sciences Centre, Manchester, UK; 2 Department of Diabetes and Endocrinology, Salford Royal Hospital, Salford, UK; 3 University Psychiatric Center, Leuven, KU Belgium; 4 Res Consortium, Research, Andover, USA; 5 Mersey Deanery Psychiatry Rotation, Manchester, UK; 6 Department of Medicine, Kings Mill Hospital, Mansfield, UK; 7 Institute of Psychiatry, Pharmacy, London, UK

**Keywords:** Antipsychotic, Prescribing, Variations, General practice, Demographic, Psychosis

## Abstract

**Background:**

Antipsychotic medications are the first-line pharmacological intervention for severe mental illnesses (SMI) such as schizophrenia and other psychoses, while also being used to relieve distress and treat neuropsychiatric symptoms in dementia.

Our aim was to examine the factors relating to antipsychotic prescribing in general practices across England and how cost changes in recent years have impacted on antipsychotic prescribing.

**Methods:**

The study examined over time the prescribing volume and prices paid for antipsychotic medication by agent in primary care.

Monthly prescribing in primary care was consolidated over 5 years (2013–2018) and DDD amount from WHO/ATC for each agent was used to convert the amount to total DDD/practice.

The defined Daily Dose (DDD is the assumed average maintenance dose per day for a drug used for its main indication in adults.

**Results:**

We included 5750 general practices with practice population > 3000 and with > 30 people on their SMI register. In 2018/19 there were 10,360,865 prescriptions containing 136 million DDD with costs of £110 million at an average cost of £0.81/DDD issued in primary care.

In 2017/18 there was a sharp increase in overall prices and they had not reduced to expected levels by the end of the 2018/19 evaluation year. There was a gradual increase in antipsychotic prescribing over 2013–2019 which was not perturbed by the increase in drug price in 2017/18.

The strongest positive relation to increased prescribing of antipsychotics came from higher social disadvantage, higher population density (urban), and comorbidities e.g. chronic obstructive pulmonary disease (COPD). Higher % younger and % older populations, northerliness and non-white (Black and Minority Ethnic(BAME)) ethnicity were all independently associated with less antipsychotic prescribing.

Higher DDD/general practice population was linked with higher proportion(%) injectable, higher %liquid, higher doses/prescription and higher %zuclopenthixol depot. Less DDD/population was linked with general practices using higher % risperidone and higher spending/dose of antipsychotic.

**Conclusions:**

The levels of antipsychotic prescribing at general practice level are driven by social factors/comorbidities. We found a link between depot prescriptions with higher antipsychotic DDD and risperidone prescriptions with lower antipsychotic DDD. It is important that all prescribers are aware of these drivers / links.

## Background

Antipsychotic medications are the first-line pharmacological intervention for severe mental illnesses (SMI) such as schizophrenia [1, 2] and other psychoses, while also being used to relieve distress and to treat neuropsychiatric symptoms in dementia [3]. Oral antipsychotic medication prescribed in primary care is one of the main sources of support to people with a history of psychosis.

The American Psychiatry Association (ADA) Clinical Practice guidelines [1] and NICE guidance [2] recommend treatment with second generation antipsychotic agents (SGA) first line for psychosis. However first generation antipsychotic (FGA) agents are still widely prescribed, often for historical reasons in the case of a particular individual. The medication is largely recommended by specialist teams and prescribed in general practice, with the exception of Clozapine and depot antipsychotic treatment [4].

It was shown by Marston et al. in 2014 [5] using a United Kingdom (UK) Primary Care database that of those adults receiving first-generation antipsychotics (for all forms of psychosis), less than 50% had a diagnosis of psychosis/bipolar disorder. For the second-generation agents, the numbers ranged from 4824 (36%) for quetiapine to 7094 (62%) for olanzapine. In patients without psychosis/bipolar disorder, common diagnoses included anxiety, depression, dementia, sleep and personality disorders. Thus in many cases, these agents are used for other indications than psychosis.

Since 2014 many antipsychotic medicines have moved to generic provision. In 2017/18 supplies of certain generic agents were affected by substantial price increases [6].

We have previously looked at the general practice level factors related to prescribing of antidepressants in England and show that the quality of patient-general practice relationship influences the antidepressant prescribing rate [7]. Our analysis utilised the same modelling paradigm. Our aim was to examine the factors that relate to antipsychotic prescribing in general practices across England.

## Methods

The study examined over time the amount and prices paid in antipsychotic medication by agent in primary care and considered if this affected selection of agents by prescribers. There was no a priori hypothesis - we were commenting on an observed trend.

All data was analysed at general practice level for all general practices in England. No individual patient data was accessed. We also looked at the general practice characteristics including disorder registers and local demography as potential determinants of antipsychotic prescribing. Antipsychotic agents were looked at individually. The monthly prescribing data was aggregated to the annual amounts for each year.

All psychiatric and physical comorbidities that are available at a general practice level for England were included in the regression analysis. Only those with a significant relation to the level of antipsychotic prescribing were included. There was no data about substance misuse at a general practice level.

The National Health Service (NHS) in England and Wales publishes publicly each month the prescribing in general practice by each British National Formulary (BNF) code which distinguishes between the various prescribed agents. This is freely available at https://opendata.nhsbsa.net/dataset/english-prescribing-data-epd [8]. Practice demographics (age, gender, ethnicity) were obtained from the on-line resource provided by Public Health England [9]. Details at a practice level on comorbidities were obtained from the Quality and Outcomes Framework (QOF) from NHS Digital [10].

This was aggregated for the year 2018/19 using Defined Daily doses (DDD) as published by the World Health Organisation Annual Therapeutic Classification WHO/ATC [11] and analysed by delivery method (oral/intramuscular) and dose level for agents to treat psychosis or behaviour felt to put the individual or those around them at risk.

Monthly prescribing in primary care was consolidated over 5 years and DDD amount from WHO/ATC for each agent was used to convert the amount to total DDD/practice. The DDD is the assumed average maintenance dose per day for a drug used for its main indication in adults. Cost of each agent year on year was determined [6, 11]. Analysis by the Department of Health and Social Care has identified a range of responsible factors, including loss of license by certain providers, medicine shortages, currency fluctuations, and increases in wholesalers’ margins linked to specific generic medicines, that influence the price of specific medications.

We examined 3 different classes of possible factors that could influence the antipsychotic prescribing rate.

- The local population was characterised by Location & Demographic indicators including
  Age distribution & Gender was taken from the published practice details
  Social Deprivation was taken from the Office of National Statistics published Index of Multiple Deprivation score (IMD 2015) for the general practice
  Ethnicity % Black and Minority Ethnicity (BAME) was taken from the responses received to the general practice patient survey
  Latitude (Northerliness) was taken from the postal address
  Population Density (urban/rural) was taken from Office of National statistics publication for number of population within given area for the Lower Layer Super Output Area (LSOA) or Middle Layer Super Output Area (MSOA) for larger practices containing the practice
- Practice Characteristics including general health was

- Practice Size was taken as the total patient list taken from the Quality and Outcome Framework (QOF)
- Practice level Comorbidities (Depression, Diabetes, Chronic Obstructive Pulmonary disease (COPD)) was taken from the Disease register published by the Quality and Outcome Framework (QOF)

c) Practice mental health prescribing behaviour was characterised using the following indicators

- Amount of Anxiolytic & Hypnotic Use DDD/population
- Number of different types of antipsychotic used (based on the unique number of different medicine, the dose level and method of application (including injection, tablet, liquid) combinations)
- Antipsychotic Doses/Prescription by individual agent
- Antipsychotic Average Cost / Dose by individual agent
- We applied stepwise multivariate regression analysis to establish the association of these factors to the local practice antipsychotic prescribing rate taken total antipsychotic DDD/population. The Analyse-it add in was used in conjunction with Power pivot and excel and 64-bit windows 10

## Results

We included 5750 general practices with population > 3000 and with more than 30 people on their severe enduring mental illness (SMI) register. This cut-off was taken for the total number of patients on anti-psychotic medication within the practice to reduce the outlier effect of practices with small numbers of SMI patients.

In 2018/19 there were 10,360,865 million prescriptions containing 136 million DDD with costs of £110 million at an average costs of £0.81 / DDD issued in primary care.

**Fig. 1:**
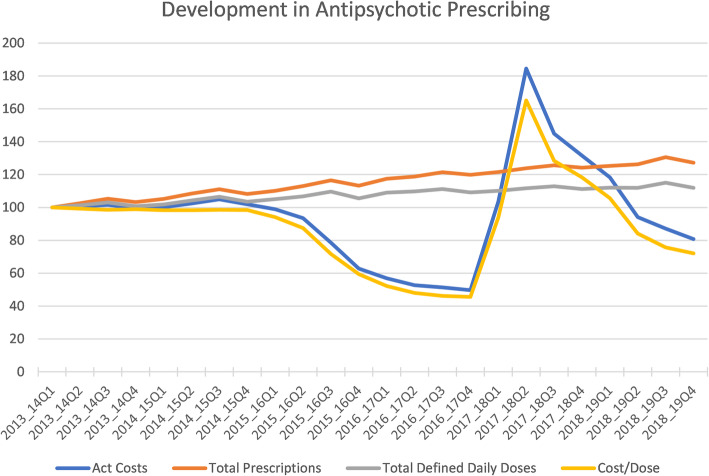
Development of antipsychotic prescribing over 5 years including changes in drug cost. Q = quarter 1, 2, 3 or 4 of the year

Figure 1 shows the progression of prescribing over a 5 year period. The unforeseen price rises in 2017/18 caused a sharp increase in overall prices and they had not reduced to the expected levels by the end of the 2018/19 evaluation year. There was a gradual increase in antipsychotic prescribing which was not perturbed by the increase in drug price in 2017/18. The changes in price and volume are given in Table 1.

**Table 1:** Change in price and volume 2015/16 to 2018/19 for those antipsychotics with the greatest change in volume of prescribing

	Actual Cost/DDD 2018/19 as % 2015/16 cost	Change in Total Prescriptions 2015/16 to 2018/19	Number of Prescriptions 2018/19
Quetiapine	123%	686,003	3,456,355
Olanzapine	326%	180,762	2,386,096
Risperidone	140%	68,435	1,720,600
Aripiprazole	15%	307,222	1,100,913
Amisulpride	135%	2424	393,944
Haloperidol	386%	−15,159	331,199
Chlorpromazine	1438%	− 68,179	264,164

### General practice characteristics vs antipsychotic prescribing

**Fig. 2:**
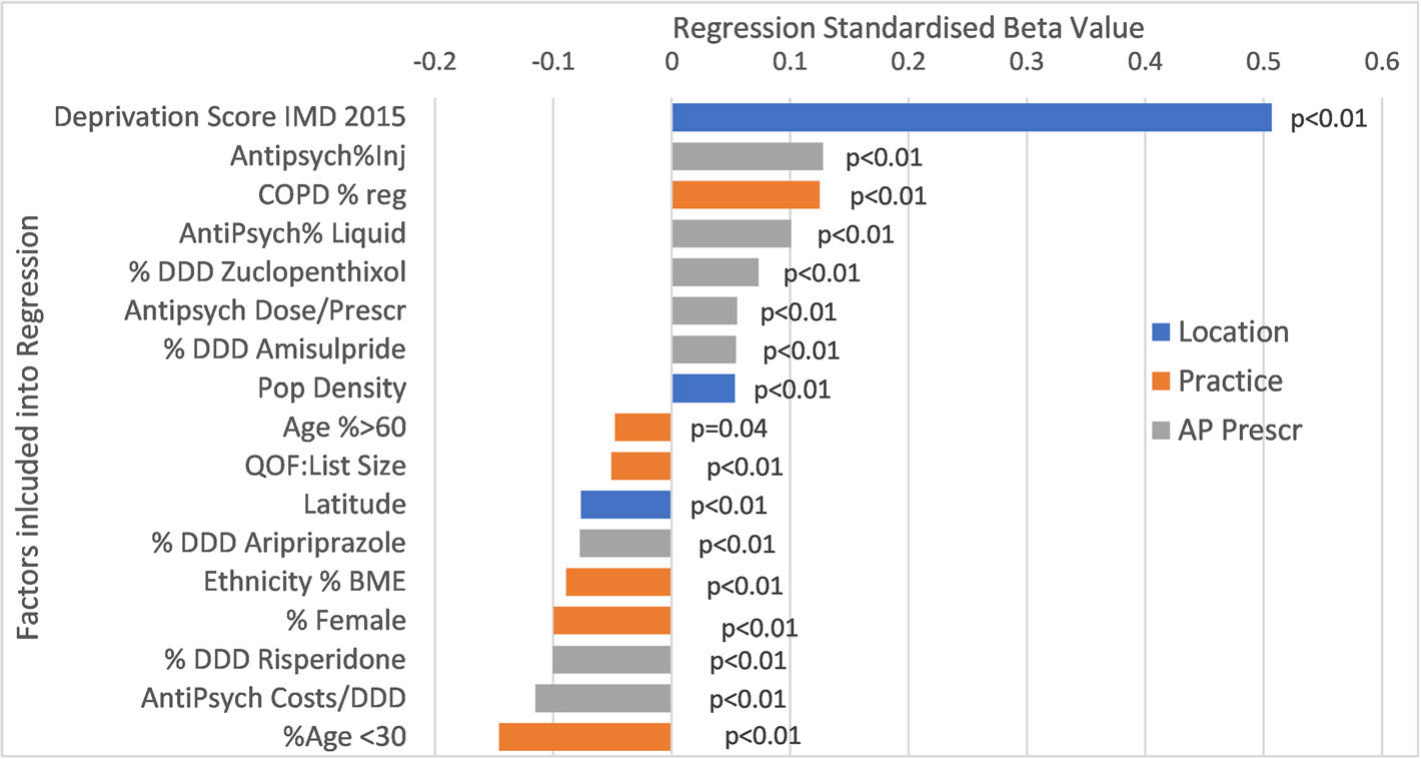
Multivariate regression describing the factors at a general practice level relating to antipsychotic prescribing. The length of the bars in Fig. 2 relates to the size of the standardised beta. The defined Daily Dose (DDD is the assumed average maintenance dose per day for a drug used for its main indication in adults. This is specific for each agent shown here

Figure 2 shows the results of the regression analysis (r^2^ = 17.4%). The strongest positive relation to increased prescribing of antipsychotics DDD/practice list came from higher social disadvantage, higher population density (urban), and comorbidities for example chronic obstructive pulmonary disease (COPD), while less antipsychotic prescribing was associated with higher % younger and % older populations, northerliness and higher proportion of non-white (Black and Minority Ethnic (BAME)) ethnicity.

### Prescribing factors vs antipsychotic prescribing

Higher antipsychotic prescribing DDD/population was linked with specifically higher % injectable, higher % liquid, higher doses/prescription and % zuclopenthixol depot use, while less antipsychotic DDD/population was linked with general practices using specifically higher % risperidone and higher spending/dose of antipsychotic.

## Discussion

The levels of antipsychotic prescribing at general practice level in the UK are associated with social factors and with comorbidities. The regression analysis that we undertook indicated that particular drivers are high population density and socioeconomic deprivation. Furthermore transcultural factors (BAME ethnicity associated with less antipsychotic prescribing) need to be considered in relation both treatment of psychosis [12]. This relates to the fact in our view that BAME ethnicity people are less likely to see antipsychotic treatment as a way forward for them in terms of management of psychosis.

The influence of ‘northerliness’ is intriguing. We speculate that the northerliness or latitude of a practice has a number of possible influences such as a) climate as the levels of rain/light changes b) wider cultural behaviour & values; people in the North of England can hold different lifestyle/cultural views than those living in the South of England. However more work is required to confirm / refute these suggestions.

There have been significant fluctuations in the cost of many antipsychotic preparations in the last 3 years and these rapid changes can put pressure on clinical decision making, with impact on patient outcomes with regards to stability of mental state over time. Interestingly however, despite the price fluctuation, we saw a consistent steady rise in antipsychotic prescribing over the 5 year period (2013–2018) that we examined. This was also reported by Roberts et al. in 2019 [13]. They described a consistent increase in the proportion of atypical antipsychotics prescribed, compared to typical antipsychotics, between 2007 and 2014, with atypicals accounting for 79.9% of total antipsychotics prescribed in 2014 in England and Wales.

The data that we have accessed only covers family doctor prescriptions. In the UK, initiation of antipsychotic prescriptions is generally done by psychiatrists. It should be pointed out that the data for England is generalisable to the UK as a whole in that all 4 nations of the UK broadly adhere to the same guidance.

In the Marston et al. study [5] in a large primary care database representative of the UK, approximately half of the prescriptions for first-generation and second-generation antipsychotics are issued to people who have no record of SMI, defined as schizophrenia, bipolar affective disorder or other non-organic psychosis in their clinical notes. Furthermore, they were more likely to be prescribed to older people who may be more sensitive to adverse effects such as movement disorders and cardiometabolic risk [14, 15]. As shown by Szczepura et al., in 2016 [16], individuals with dementia are sometime treated with antipsychotics in order to reduce/attenuate challenging behaviour. When antipsychotics are prescribed to people without SMI, they tend to be given in lower doses and for slightly shorter periods, with the exception of people with ADHD and dementia who receive these drugs for relatively long periods. In our study, we were not able to examine dose prescribed by specific condition.

While the growth in generic medication brings substantial cost advantages it also brings significant risks in supply and cost forecasting which may affect the recommendations made by medicines management committees [17].

The association between lower overall antipsychotic prescribing vs specifically risperidone prescribing, and high overall prescribing vs specifically depot antipsychotic prescribing is a relevant finding. The development of receptor supersensitivity in relation to long-term exposure to first generation antipsychotic agents was described in a seminal paper by Prien in 1969 [18]. Whether this is a contributory phenomenon here is not something that our methodology can address.

General practice (family doctor) prescribing in England and Wales is generally in accordance with National Institute for Clinical Excellence (NICE) guidance [2] in relation to antipsychotic prescribing for psychosis and behavioural disturbance. Depot medication is still prescribed for many patients.

Further database work could explore side-effects associated with these antipsychotic prescriptions, and the treatment decisions pre-dating the choice of an antipsychotic agent.

### Strength/weaknesses

We have analysed national prescribing data over a 5 year period. However this is at general practice not individual level. Nevertheless that can enable visibility of trends that would be more difficult to elucidate at individual patient level. Furthermore we did not have access to hospital initiated prescriptions for this analysis.

## Conclusion

General practice (family doctor) prescribing in England and Wales is generally in accordance with National Institute for Clinical Excellence (NICE) guidance [2] in relation to antipsychotic prescribing for psychosis and behavioural disturbance. Depot medication is still prescribed for many patients.

It needs to be recognised that the level of antipsychotic prescribing at a general practice level are driven by social changes and comorbidities. Despite the price fluctuations, we saw a consistent steady rise in antipsychotic prescribing over the 5 year period (2013–2018) that we examined.

## Data Availability

The analysis used publically available general practice level data. Specifically the National Health Service (NHS) in England and Wales publishes publicly each month the prescribing in general practice by each British National Formulary (BNF) code, which distinguishes between the various prescribed agents. This is freely available at https://opendata.nhsbsa.net/dataset/english-prescribing-data-epd. Practice demographics (age, gender, ethnicity) were obtained from the on-line resource provided by Public Health England. Details at a practice level on comorbidities were obtained from the Quality and Outcomes Framework (QOF) from NHS Digital.

## Abbreviations

ADA: American Psychiatry Association
BAME: Black and Minority Ethnicity
BNF: British National Formulary
COPD: Chronic Obstructive Pulmonary disease
DDD: Defined Daily doses
FGA: First generation antipsychotic
IMD: Index of Multiple Deprivation score
LSOA: Lower Layer Super Output Area
MSOA: Middle Layer Super Output Area
NHS: National Health Service
NICE: National Institute for Clinical Excellence
QOF: Quality and Outcome Framework
SMI: Severe mental illnesses
UK: United Kingdom
WHO/ATC: World Health Organisation Annual Therapeutic Classification
